# ‘Jump start’ childcare-based intervention to promote physical activity in pre-schoolers: six-month findings from a cluster randomised trial

**DOI:** 10.1186/s12966-020-0910-6

**Published:** 2020-01-16

**Authors:** Anthony D. Okely, Rebecca M. Stanley, Rachel A. Jones, Dylan P. Cliff, Stewart G. Trost, Donna Berthelsen, Jo Salmon, Marijka Batterham, Simon Eckermann, John J. Reilly, Ngiare Brown, Karen J. Mickle, Steven J. Howard, Trina Hinkley, Xanne Janssen, Paul Chandler, Penny Cross, Fay Gowers

**Affiliations:** 1 Early Start, Faculty of Social Sciences, University of Wollongong, Northfields Ave, Wollongong, New South Wales 2522 Australia; 2 Institute of Health and Biomedical Innovation at Queensland Centre for Children’s Health Research, School of Exercise and Nutrition Science, Queensland University of Technology, Brisbane, Queensland Australia; 3 School of Early Childhood, Queensland University of Technology, Kelvin Grove, Queensland 4059 Australia; 4 Institute for Physical Activity and Nutrition (IPAN), School of Exercise and Nutrition Sciences, Deakin University, Geelong, Australia; 5 School of Psychological Science and Health, University of Strathclyde, Glasgow, Scotland UK; 6 Institute of Health and Sport, Victoria University, Melbourne, Australia

**Keywords:** Physical activity, Children, Early childhood, Randomised controlled trial, Sedentary behaviour, Disadvantage

## Abstract

**Background:**

Participation in adequate levels of physical activity during the early years is important for health and development. We report the 6-month effects of an 18-month multicomponent intervention on physical activity in early childhood education and care (ECEC) settings in low-income communities.

**Methods:**

A cluster randomised controlled trial was conducted in 43 ECEC settings in disadvantaged areas of New South Wales, Australia. Three-year-old children were recruited and assessed in the first half of 2015 with follow-up 6 months later. The intervention was guided by Social Cognitive Theory and included five components. The primary outcome was minutes per hour in total physical activity during ECEC hours measured using Actigraph accelerometers. Intention-to-treat analysis of the primary outcome was conducted using a generalized linear mixed model.

**Results:**

A total of 658 children were assessed at baseline. Of these, 558 (85%) had valid accelerometer data (mean age 3.38y, 52% boys) and 508 (77%) had valid accelerometry data at 6-month follow-up. Implementation of the intervention components ranged from 38 to 72%. There were no significant intervention effects on mins/hr. spent in physical activity (adjusted difference = − 0.17 mins/hr., 95% CI (− 1.30 to 0.97), *p* = 0.78). A priori sub-group analyses showed a greater effect among overweight/obese children in the control group compared with the intervention group for mins/hr. of physical activity (2.35mins/hr., [0.28 to 4.43], *p* = 0.036).

**Conclusions:**

After six-months the Jump Start intervention had no effect on physical activity levels during ECEC. This was largely due to low levels of implementation. Increasing fidelity may result in higher levels of physical activity when outcomes are assessed at 18-months.

**Trial registration:**

Australian New Zealand Clinical Trials Registry  ACTRN12614000597695.

## Background

Participation in adequate levels of physical activity during early childhood (defined here as ages birth to 5 years) is beneficial to a child’s health and development [1]. Adequate levels have been defined in national guidelines from a number of countries as at least 180 min per day of physical activity of any intensity [2, 3]. Many children in Australia do not meet these guidelines, especially those living in low socio-economic communities [4]. Finding ways to increase and maintain the number of young children who are adequately active is needed.

Early childhood education and care (childcare) settings are important for promoting physical activity, especially in low-income communities. Most early childhood curriculum standards and frameworks mandate the provision of physical activity opportunities for children whilst attending childcare settings [5]. Several systematic reviews have been inconclusive in showing that childcare-based interventions are efficacious in increasing physical activity levels among children while at childcare [6, 7]. This may be due to poor study quality, and low levels of implementation fidelity, especially when interventions are being implemented by childcare staff. This latter point may be due to staff not receiving an adequate “dose” of professional development to successfully integrate and sustain change into their daily routines [8, 9]. These reviews also show that there is less evidence in low-income communities; only six studies were reported and only one of these found a significant effect [8]. Further, this effect was from a short-term intervention (3 months duration). There are very few studies in these communities with follow-ups longer than 12 months. Other evidence gaps identified were reporting intervention results for sub-groups such as boys and girls and overweight/obese and healthy weight children.

Given the lack of evidence on efficacious interventions in childcare settings in low-income communities, the primary aim of this study was to test an 18-month multi-component, multi-setting intervention for promoting physical activity among pre-school-aged children in these settings. As intervention effects may be moderated by a child’s sex, age, initial physical activity level and adiposity, a secondary aim was to test if the intervention results differed between boys and girls, younger and older and overweight and non-overweight children, and children who were adequately or inadequately active. Sub-group analyses for sex, age, baseline physical activity level and baseline body mass index (BMI) were also reported. This paper reports the 6-month results from this study.

## Methods

### Study design and setting

*Jump Start* was a multi-component, multi-setting childcare-based cluster randomised controlled trial. Assessments were conducted at baseline, 6 months and 18 months. The study was conducted across the state of New South Wales, Australia. The sampling frame comprised childcare centres located in areas of disadvantage in NSW, Australia, according to the area location indices for socio-economic disadvantage (SEIFA) [10]. The Study followed the Consolidated Standards of Reporting Trials (CONSORT) Statement [11] and Extension to Cluster Randomised Trials [12]. The study was registered with the Australian New Zealand Clinical Trials Registry (ACTRN12614000597695) and approved by the University of Wollongong Human Research Ethics Committee (HE14/137). Parents of participants provided written informed consent for their child to participate in the study. If a parent of a child in an intervention centre did not provide written informed consent, their child still participated in the intervention but not in the data collection. The study protocol has been previously published [13].

### Participants and recruitment

#### Childcare Centres

Centres were eligible to participate in the study if they were located in an area with a SEIFA index of relative socioeconomic disadvantage of less than or equal to 5 (lowest 50%) and had a minimum enrolment of five eligible consenting children. Recruitment of centres occurred from January 2015 to June 2015.

#### Children and staff

Children were eligible to participate in the evaluation components of the study if they were 3 years old or turning three before the start of the intervention; attended at least 2 days a week at a participating childcare centre; and were not likely to be enrolled in primary school the following year. All parents/caregivers of eligible children received a participant information sheet and a link to an online recruitment video designed to simplify the explanation of the study. All staff working with 3-year-olds in the ECEC centres were also invited to participate in the study.

### Randomisation and allocation

Following recruitment and baseline testing, centres were pair-matched according to the number of staff and children in attendance, geographical location and Indigenous status of the centre. Centres within each pair were then randomised to the Jump Start intervention or usual practice comparison group by the project statistician (MB), who was not involved in the recruitment or intervention delivery, using a concealed computerised random number generator.

### Intervention

The *Jump Start* intervention comprised five components as described in Table 1. These were designed to complement one another and provide multiple opportunities to integrate physical activity into the daily routine of the centre. The intervention was based on Bandura’s Social Cognitive Theory (SCT) and focused on the personal, behavioural and environmental factors that influence physical activity participation in childcare settings. The intervention was developed using the “working backwards” process developed by Robinson and reported in previous interventions [14, 15]. The explicit links to the Australian ECEC sector’s framework and curricula (National Quality Standard and Early Years Learning Framework) [5, 16] were made.

**Table 1:** A description of the five components of the *Jump Start* intervention

Jump start component	Description of component	Who facilitates the component
Jump in	Structured gross motor lessons, which will be facilitated every day for approximately 20 min. This component focuses on one gross motor skill, across two lessons every fortnight for 13 skills. All skill lessons are repeated three times over the 18-month period. The skill experiences are based on fun, interactive and engaging games.	Educators
Jump out	Provision of opportunities for children to practice the gross motor skills taught in the Jump In component every day. It provides opportunities for educators to engage with the children in physical activity and encourage the correct performance of the skills. Jump Out is predominantly child-led and educators respond to the child’s cues using a variety of intentional teaching methods.	Educators
Jump up	Music-based activities designed to break up long periods of sedentary behaviour with high-energy physical activity. The children and educators will engage in two 3-min songs every day.	Educators
Jump through	Activities designed to connect learning and movement. This component aims to use movement to enhance the learning experience. This component will be facilitated twice a day using a range of fun and engaging strategies.	Educators
Jump home	Opportunities provided to families to learn about Jump Start and for parents/caregivers to participate in the same activities at home that the children have been participating at the ECEC centre.	Parents/caregivers

#### Staff training for the intervention group

The intervention was designed to be implemented by all staff in a childcare centre. Professional development (PD) for staff in each centre was delivered by trained childcare educators through an intensive one-day PD session and ongoing bespoke PD opportunities provided during the intervention period. The one-day intensive PD involved 7 h of face-to-face contact or virtual contact through videoconferencing technology, and covered background information and the philosophy behind the intervention, reflection on current practices, content related to each component, opportunities to experience and practice delivery of each component, and a reflection on how the intervention could be integrated in the daily routines at the centres. Ongoing bespoke PD was also available to all staff, which focused on additional training in the specific components of the intervention. Each centre received one dedicated support visit in the first 6 months of the intervention. Within this time frame there was also a process evaluation visit where additional support was provided after process data had been collected.

### Control group

For many centres allocated to the control group, they continued to implement the *Munch & Move* program [17]. *Munch & Move* is an initiative of the NSW Government and provided, from 2010 to 2015, free face-to-face training for one staff member on promoting healthy eating, active play and fundamental movement skills and reducing screen time in their centre. From 2016, Munch and Move offered online professional learning and support through health promotion officers from the local area health service. In this study, we sent regular emails to staff in control centres encouraging them to take up the online *Munch & Move* training and to contact the Department of Health if they required any resources. A truncated version of the *Jump Start* intervention was offered to staff at the end of the full intervention period (18-months).

### Data collection procedures

Data were collected by trained research assistants blinded to group allocation. Baseline data were collected from February–June 2015, and 6-month follow-up data collected from the same cohort of children from August–December 2015. Only physical activity data were collected 6-months into the trial. Although this was an 18-month intervention, we chose to collect and report data at 6-months as we believed it would provide a long enough intervention period to avoid any novelty effects (overcome threats to internal validity of the study) and was potentially long enough to have an identifiable effect on the primary outcomes. That is, we wanted to determine if intervention effects could be seen as early as 6 months after baseline. To ensure all data collectors remained blinded during the assessment periods, they conducted assessments either in the intervention or control centres only. Staff were asked not to discuss group allocation with data collectors. In addition, the objectively assessed primary outcome measure was selected to minimise the potential for bias.

### Measures

#### Outcome measures: physical activity levels

Physical activity was measured using Actigraph accelerometer models GT1M, GT3X+ and GT3X models which display high levels of agreement [18]. Minutes per hour spent in total physical activity (a combination of light-, moderate-, and vigorous-intensity physical activity; LMVPA) while at the childcare centre was the primary outcome. Other physical activity outcomes included minutes per hour spent sedentary and in moderate- to vigorous-intensity physical activity (MVPA). Children were asked to wear an accelerometer for 1 week during waking hours, except during water-based activities. Collected accelerometer data were integrated into 15 s epochs during data reduction. After screening for non-wear periods (≥20 min of consecutive ‘0’ counts), participant data were considered valid at each time point if they accumulated ≥3 h of valid wear time during childcare centre hours on ≥1 childcare day. These criteria were chosen because: i) 3 h represented 40–43% of a typical childcare day (7.0–7.5 h), and ii) this study was a group RCT and, as such, the aim was to represent total physical activity at the centre level from individual child samples. Therefore, less stringent inclusion criteria (e.g., ≥ 1 day) were acceptable because these errors may not bias centre-level estimates, and loss of precision may be overcome by increasing sample size [19]. Epochs recording ≥200 counts/15 s were classified as LMVPA [20] and epochs ≥420 counts/15 s and ≤ 25 counts/15 s during childcare hours were classified as MVPA and sedentary behavior, respectively [21, 22].

#### Weight status

Weight status was evaluated by measuring height and weight and calculating body mass index following standardized protocols [23].

#### Demographic characteristics

Demographic information was collected on the staff, parents/caregivers and participating children. Demographic variables included child date of birth, sex, Aboriginal status and ethnicity. Parents/caregivers reported their age, sex, postcode, marital status, education status, employment status, gross annual income, Aboriginal status, ethnicity and family structure. Childcare staff reported their age, sex, qualifications, years of experience (in childcare and in the participating centre), and level of training, experience, and self-efficacy in physical activity and motor skill development. Socio-economic status (SES) for children was based on their postcode of residence using the Australian Bureau of Statistics census-based SEIFA scores.

#### Process measures

Intervention fidelity was assessed by trained research assistants on one occasion over a one-day period using a study-specific direct observation instrument [24]. The instrument recorded start and finish times for each Jump Start component (see Table 1), the number of children (3–5 year-olds) involved in each component, adherence to the structured lesson plans (where appropriate), description of activities, use of equipment and resources, staff behaviours, and additional comments (e.g., weather and environmental changes).

For the Jump Home component, parents were asked to complete a checklist each week documenting the number of activities completed. This checklist was asked to be returned to the child’s centre or send directly to the research team.

For the 21 comparison centres, a direct observation tool was developed to measure implementation of the Munch & Move program. This instrument consisted of 62 “yes” or “no” and open ended questions and focused on types structured physical activity lessons (12 questions), unstructured physical activity or gross motor experiences (6), educator intentionality (5), equipment and resources available which promoted physical activity (7), intentional energy breaks (4), activity levels in routine activities (16), and communication strategies used by educators with families regarding physical activity and gross motor experiences (2). The questions incorporated specific physical activity measures such as, minutes of activity and proportion of children involved. Educator behaviours, including educators asking questions relating to physical activity experiences or educators providing positive prompts/statements in relation to physical participation with the children were also recorded.

The questions were categorised into four components: 1) structured physical activity, 2) unstructured physical activity, 3) staff intentionality, and 4) resources and equipment. Each centre received a score for each component which comprised minutes of structured and unstructured physical activity, number of children involved, variability of equipment, staff intent to promote a physically active environment and their communication to parents. Components were coded as 1, 2 or 3 (1 = low, 2 = average, 3 = high) by using the mean and standard deviation. A low scoring component was one below the mean value, an average score was the mean and a high score was above the total of the mean and half a standard deviation value. Summing each component then provided all centres with an overall score (minimum score = 0, maximum score = 12).

### Sample size calculations

Sample size and power estimates were based on the formula proposed by Murray [25] to adjust for a clustered (nested) cohort design. Based on the changes observed in our pilot studies for accelerometer-based physical activity, we estimated the minimum acceptable difference between groups to be 45 mins/day of total physical activity (LMVPA) at the 18-month time point which translated to an effect size (Cohen’s d) of 0.4 [26]. For a two-tailed alpha = 0.05 and an intraclass correlation (ICC) of 0.01–0.05 our proposed sample size of 608 participants (304 per group) provided approximately 86% power to detect an intervention effect of 0.4 or greater for the ICC range proposed.

### Statistical analyses

Analyses were conducted using STATA (Version 15, StataCorp LCC, College Station TX) following the procedures as outlined in Murray [25]. Summary statistics were created for the variables of interest (child sex, BMI, and activity level) and accelerometer wear time. T-tests or chi-square tests were used to determine if students who provided data at 6-month follow-up differed to those that only provided baseline data on the following characteristics: sex, baseline age, weight status and physical activity level. Significance levels were set at *p* ≤ 0.05. As there was a large amount of missing data at baseline (due mostly to monitors not worn outside of child care attendace) and the focus of the intervention was on promoting physical activity during childcare hours, a decision was made to only assess physical activity during childcare hours for the 6-month follow-up data collection. The primary outcome remained the same (time spent in LMVPA) but was operationalised as minutes per hour (mins/hr) during childcare to allow for different operating hours of centres and child time of attendance, which was determined from the attendance logbook kept by each centre.

#### Changes in physical activity

Analyses followed intention-to-treat principles. Analysis of the primary outcome (minutes per hour in total physical activity [LMVPA]), and the other physical activity outcome variables (mins/hour sedentary and in MPA, VPA and MVPA) was conducted using a general linear mixed model (GLMM) which contained random effects for time and childcare centre nested within group.

#### Sub-group analyses

As child sex, age and weight status are common moderators of physical activity interventions [27], sub-group analyses were performed comparing boys and girls, children’s baseline BMI (categorised into two groups: ‘underweight/healthy weight’ and ‘overweight/obese’ based on the IOTF cut-points [28]), and age (categorised based on a median split). We included moderator interaction terms in the above GLMM separately for all potential moderators and presented the results by mediator subgroup if the test for three-way interaction term (group x time x moderator) was significant at the non-conservative 20% threshold [29].

#### Process measures

Observation data were presented as a percentage of intended components completed (see Table 1 for components). Each component was evenly weighted out of 25%. Zero was given if no Jump Ups were observed, 12.5% if only one Jump Up was observed and 25% was if ≥2 Jump Ups were observed. A similar scoring system was utilised for the Jump Out and Jump Through components. For the Jump In component, the structured lessons comprised four components and these were scored based on their relative importance to provide a total score of 100, which was then standardised to a score out of 25%.

## Results

### Sample

**Fig. 1:**
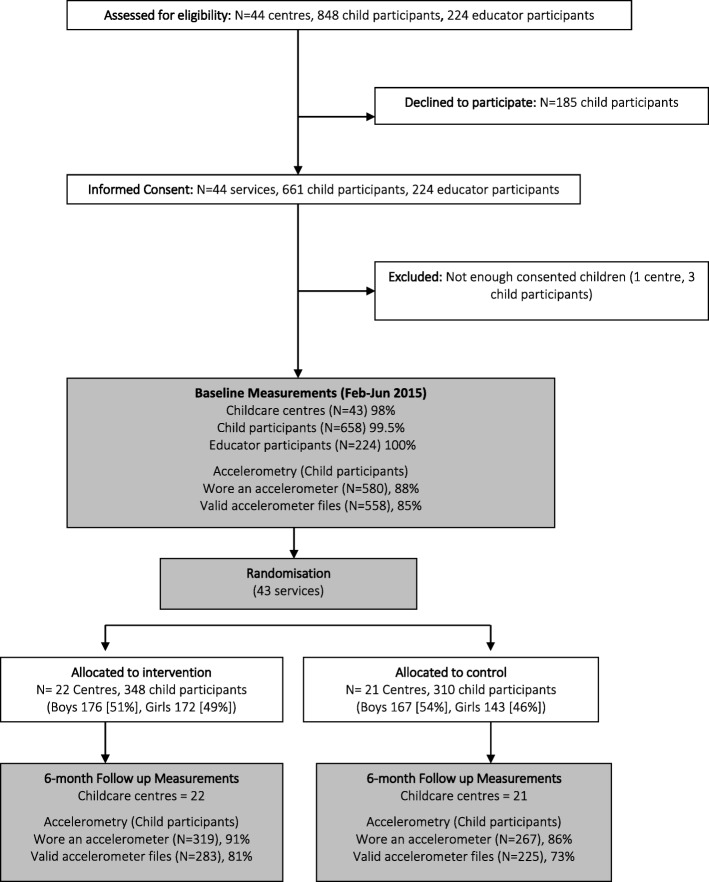
CONSORT flowchart showing progression of participants through the study

Figure 1 shows the flow of participants through the study, from recruitment to 6-months post baseline. Forty-four centres were assessed for eligibility and invited to participate. Of these, one centre did not meet the eligibility criteria due to not having the minimum number of consenting children (*n* = 3) resulting in 43 childcare centres participating in the study. Parental consent was received from 661 of the 848 (78%) children eligible for the study. Baseline characteristics of the 658 children who were assessed are outlined in Table 2. Ninety-six percent of the 580 children who wore an accelerometer provided at least 1 day of valid accelerometer data (558/580). At 6-months, 586 children wore an accelerometer and 87% of these provided at least 1 day of valid accelerometer data (508/586). There were no significant differences in the proportions of boys and girls (83% vs 81%, χ^2^ = 0.891, *p* = 0.345), baseline BMI (16.5[1.4] vs 16.4[1.5] kg/m^2^, *p* = 0.644), age (3.3[0.4] vs 3.3[0.3] y, *p* = 0.141), or baseline activity level (15.35[4.05] vs 15.13[4.32)]min/hr., *p* = 0.596) between those who provided follow up data compared with those who did not.

**Table 2:** Baseline characteristics of the sample

Characteristic	Intervention Group	Control Group
Number of children	348	310
Boys	176 (50.6%)	167 (53.9%)
Girls	172 (49.4%)	143 (46.1%)
With valid accelerometer data	298 (85.6%)	260 (83.9%)
Mean age (years) and SD	3.42 (0.35)	3.34 (0.35)
Aboriginal or Torres Strait Islander (%)	79 (22.7%)	95 (30.6%)
Child BMI Category (%)		
Underweight	19 (5.5%)	13 (4.2%)
Healthy weight	266 (76.4%)	235 (75.8%)
Overweight	47 (13.5%)	32 (10.3%)
Obese	6 (1.7%)	9 (2.9%)
Mean number of days/week child attends childcare	2.76 (0.96)	2.70 (0.95)
Mean number of hours/week child attends childcare	19.44 (9.24)	20.09 (9.40)
Child activity level		
Active (≥15mins/hour LMVPA while at childcare)	162 (54.5%)	130 (49.8%)
Inactive (<15mins/hour LMVPA while at childcare)	135 (45.5%)	131 (50.2%)
Number of educators who completed demographic questionnaire	160 (97.6%)	93 (76.2%)
Number of educators who completed the self-efficacy survey	149 (90.9%)	110 (90.2%)
Education level of educators (%)		
High School	6.2	13.0
Certificate or Diploma	60.0	48.3
University trained	32.5	38.7
Question not completed	1.3	0
Mean age (years) and SD of educators	35.35 (10.88)	35.48 (12.75)
SES (from 536 Parent Surveys – 295 Intervention, 241 Control) Low SES (SEIFA Decile 1–5)	213 (72.2%)	204 (84.6%)
IRSD (from 536 Parent Surveys – 295 Intervention, 241 Control)		
< 927	73 (24.7%)	90 (37.3%)
927–965.8	67 (22.7%)	70 (29.0%)
965.8–1001.8	76 (25.8%)	48 (19.9%)
> 1001.8	79 (26.8%)	33 (13.7%)
Accelerometer wear time		
Mins per day	452	420

Just over 90% of the educators who worked in the centres completed the self-efficacy and demographics questionnaire. The mean age of educators was 35.39y (±11.58) and slightly more than one-third of educators had a tertiary qualification (Table 2). Educators also reported low levels of efficacy around teaching gross motor skills and providing physical activity experiences. A total of 117 educators attended the one-day intensive PD session. During the first 6 months of the intervention, all 18 centres received at least one support visit from the research team and 4 centres received a second support vist.

### Changes in the physical activity outcomes

Baseline and 6-month physical activity outcomes for children in the intervention and control centres are shown in Table 3. The adjusted differences between children in the intervention and control centres were not statistically significant for any of the physical activity outcomes assessed. There were no adverse events or side-effects reported.

**Table 3:** Changes in physical activity from baseline to 6-month follow-up^1^

Outcome (mins/hr)	Intervention			Control			I-C Adjusted mean difference (95% CI)^6^	Group x time *p* value
	Baseline Mean (SE)	6-months Mean (SE)	P value	Baseline Mean (SE)	6-months Mean (SE)	P value		
Total Physical Activity (LMVPA)^2^	15.34 (0.32)	16.46 (0.33)	< 0.001	15.24 (0.34)	16.62 (0.36)	< 0.001	−0.28(−1.43, 0.88)	0.639
Sedentary	28.78 (0.43)	27.27 (0.43)	0.002	29.06 (0.45)	27.10 (0.47)	< 0.001	0.44(− 0.91, 1.80)	0.521
MPA^3^	5.74 (0.15)	6.22 (0.15)	0.015	5.67 (0.16)	6.25 (0.16)	< 0.001	−0.10(− 0.62, 0.43)	0.735
VPA^4^	2.01 (0.09)	2.17 (0.10)	0.082	2.01 (0.10)	2.17 (0.10)	0.075	0.03(−0.29, 0.35)	0.867
MVPA^5^	7.74 (0.21)	8.41 (0.22)	< 0.001	7.68 (0.23)	8.42 (0.24)	< 0.001	−0.07(−0.84, 0.70)	0.861

^1^Analysis based on 558 and 508 children with at least 1 day of valid accelerometer data at baseline and 6-month follow-up, respectively

^2^LMVPA: Light-, moderate-, and vigorous-intensity physical activity

^3^Moderate-intensity physical activity

^4^Vigorous-intensity physical activity

^5^Moderate- to vigorous-intensity physical activity

^6^Adjusted for time and childcare centre nested within group

### Changes in physical activity from baseline to 6-months by sub-groups (sex, age, and baseline weight status)

The three-way interaction terms for the moderators baseline age (*p* = 0.156) and BMI category (*p* = 0.110) were below the threshold of *p* = 0.20 and therefore subgroup analyses were conducted for the primary outcome (total physical activity at childcare). Although the interaction term for sex was > 0.20 (*p* = 0.282) a decision was made a priori to conduct sub-group analyses for males and females. The 6-month physical activity results by sub-group are reported in Table 4. A greater effect was found in overweight/obese children in the control group compared with the intervention group for total physical activity (2.37 mins/hr. [− 4.58 to − 0.16], *p* = 0.036). No differences between groups for underweight/healthy weight were found at 6-month follow-up. The differences between intervention and control groups for boys or girls or younger or older children were small and not statistically significant.

**Table 4:** Changes in physical activity from baseline to 6-months by sub-groups (sex, baseline weight status, and age)

Outcome (mins/hr) HLMVPA^1^	Intervention			Control			I-C Adjusted mean difference (95% CI)^2^	Group x time p value
	Baseline Mean (SE)	6-months Mean (SE)	P value	Baseline Mean (SE)	6-months Mean (SE)	P value		
Sex								
Male	16.32 (0.45)	17.17 (0.46)	0.136	16.13 (0.46)	17.64 (0.48)	0.001	−0.66 (−2.19, 0.87)	0.398
Female	14.29 (0.40)	15.65 (0.41)	0.003	14.23 (0.43)	15.27 (0.46)	0.086	0.31 (−1.17, 1.80)	0.677
BMI category								
Underweight/healthy	15.18 (0.36)	16.35 (0.37)	0.016	15.17 (0.38)	16.38 (0.40)	0.001	−0.04 (−1.37, 1.28)	0.949
Overweight/Obese	16.58 (0.69)	16.91 (0.70)	0.509	15.99 (0.74)	18.69 (0.84)	0.027	−2.37 (−4.58, −0.16)	0.036
Age Category								
< 3.37	15.12 (0.42)	15.31 (0.43)	0.579	14.44 (0.42)	16.14 (0.44)	< 0.001	−1.48 (−3.06, 0.09)	0.065
> =3.37	15.58 (0.40)	17.48 (0.41)	0.001	16.20 (0.45)	17.23 (0.48)	0.063	0.98 (−0.65, 2.62)	0.238

^1^HLMVPA: high-light-, moderate-, and vigorous-intensity physical activity

^2^Adjusted for time and childcare centre nested within group

### Process evaluation

Levels of implementation varied considerably across the 22 intervention centres. For the four components of the intervention that were able to be monitored (Jump In, Jump Out, Jump Up, and Jump Through – see Table 1 for details), implementation ranged from 0 to 100% for all components except Jump In, which ranged from 0 to 90%. The mean levels of implementation were highest for the Jump Through (72%) and Jump Up (64%) components, respectively, and lowest for the Jump In (38%) and Jump Out (45%) components. Median levels were higher for each component except Jump Up and were reported due to the high level of variability within centres: Jump Through (100%) and Jump Up (50%) Jump In (40%) and Jump Out (50%).

The Jump Home component was not well supported by centres. Parents did not always receive the information on the specific activities covered by the educators each week. This resulted in poor adherence. Only a very small number of parents (16 in total) completed the checklists to indicate how often they completed any of the ‘homework’ activities with their child. As a result, evaluation of the home intervention component was not able to be completed.

For the comparison centres, the mean score out of 12 for implementation of the Munch & Move program was 6.86 (SD 1.59). Ten of the centres were categorised as low levels of implementation, five as average, and six as high implementers.

## Discussion

This paper reports the 6-month results from a multi-component intervention conducted in childcare centres located in low socioeconomic areas in New South Wales, Australia. At 6-months (one-third of the way through the intervention), there were no differences between intervention and control centres on the physical activity or sedentary outcomes. Across the entire sample, children increased their time spent in physical activity by about 1.5mins per hour over the 6-month period, which over a typical 7–10 h day in childcare would result in between 10 and 15mins more physical activity. However, there were no significant differences in physical activity between children in the intervention and control centres.

These findings are similar to those reported in other interventions in childcare settings in low-SES communities. Of the six studies identified in a 2016 systematic review on the effectiveness of childcare-based interventions in increasing physical activity, five reported small and non-significant effects on physical activity [8]. Another large-scale study involving a similar number of centres and children from a range of SES backgrounds also reported no intervention effects for children from low-SES pre-schools [30]. Despite the challenges in conducting interventions in these settings, the preliminary results from this intervention may be interpreted as somewhat disappointing, although it may be too early to arrive at this conclusion. More work is needed to better understand how to effectively promote physical activity in childcare centres in low-SES communities.

There are several explanations for the null findings in this study. First, the level of implementation was quite low, especially for two of the components (Jump In [structured activity lessons] and Jump Out [supporting unstructured outdoor free-play opportunities]), which had mean implementation levels of < 50%. Some centres had challenges in implementing the intervention due to a high staff turnover, low levels of confidence, and competing demands on staff, especially in the area of school readiness. For example, some centres found it challenging to integrate intervention components into their daily routine, and viewed it as something additional they had to fit into their already busy daily schedule. Some staff did not agree that children needed to be taught gross motor skills, believing that they acquired these naturally as part of their growth and maturation. Some staff were also convinced that implementing energy breaks (Jump Up component) would result in children being distracted and unable to successfully transition to the next activity. As such, it took some time for the intervention to “gain traction” in the centres and become part of their daily routine. We sought to address these challenges by providing detailed informal feedback and a summative report based on the observational data collected to the centres prior to each subsequent observation session. We made available to all educators free on-going bespoke professional learning which focused on additional training in the specific components of the intervention. This also offered additional training during face-to-face support visits or more regular follow-up phone calls, to those centres with low levels of implementation.

The seven centres that were classified as having a high level of implementation during the first 6 months of the intervention, were characterised as having a large number of staff trained in the Jump Start program. As such, the responsibility for implementation was shared among staff and not the responsibility of only one or two staff members. These centres also had strong “hands-on” support for Jump Start from their Director. This leadership from the Director allowed the program to be successfully integrated into the daily routine, and gave it a higher priority among staff than in those centres with less support from the Director.

The null finding may be attributable, at least in part, to the lack of a true control group. In our opinion, it would not have been possible to recruit or retain a no treatment control group, especially given the length of the intervention (18-months). As such, we decided to compare our intervention with current best-practice in the state of NSW, the Munch & Move Program [31]. In the Munch & Move Program resources to promote healthy eating and gross motor skill competence were made freely available to all ECEC services in the state of New South Wales. Online professional development was also provided free-of-charge to educators and educators were provided with further support if needed from health promotion officers from their local area health district. While this is atypical of what occurs in other jurisdictions, there were some key differences between Jump Start and Munch & Move. While Munch & Move includes some physical activity education, it does not focus specifically on increasing physical activity (just on promoting gross motor skill competence) and does not include components from the Jump Start intervention model such as energy breaks, integration of physical activity with other learning areas, and home-based motor activities. We provided additional support for the Munch & Move Program by providing centres with access to the Munch & Move website, reminding centres of professional development opportunities, and communicating regularly through newsletters and e-blasts about the Munch & Move Program, encouraging centres to make us of the online resources. Our observational monitoring of comparison centres showed that all had large amounts of time allocated for outdoor play (some exceeding more than 5 hours per day). As such, it was going to be a challenge to see a change in the intervention group that would be significantly greater than what was already happening in comparison centres.

An important point to note is that because the levels of physical activity were already high in the intervention centres, any intervention that might replace some unstructured time outdoors with a structured activity (like the Jump In component that focused on developing gross motor skill competency) may likely result in an initial decline in children’s activity levels until the staff become confident in being able to implement the sessions with high levels of fidelity. As we did not assess gross motor skills at 6-month follow-up, it is not possible to determine if these skills improved more in the intervention centres. This is something that will be assessed at 18-month follow-up.

We also explored potential moderators of intervention effects to determine if the intervention worked for some sub-groups. There were no sub-group effects for age or sex however a significant intervention effect was found among overweight and obese children in this study, with those in the control group participating in around 2.4 mins/hr. more physical activity than those in the intervention group. Over the course of a typical day at childcare (8 h) this translates to about 19mins/day which is around 10% of the daily amount recommended in the Australian Guidelines (180mins/day) [2]. Given children in Australia attend childcare an average of 3 days per week this difference is small and greater amounts of physical activity may be needed to prevent unhealthy weight gain among overweight and obese children. The possible explanations for this are not clear given there were no differences between groups for physical activity. It may be that the overweight and obese children in the control centres participated in more physical activity outside of their time at childcare, but this was not measured at follow-up.

### Strengths and limitations

Strengths of this study include the multi-component focus of the intervention and the emphasis on enhancing rather than replacing the quality of physical activity provision. This real-world intervention was also designed to be implemented by staff, enhancing its sustainability. Other strengths include the RCT design, use of an objective measure of physical activity, focus on regional and rural communities with high proportions of vulnerable children, where the need for high-quality learning environments in childcare is greatest, and a comparison against a best-practice control rather than a no-treatment control.

It was challenging collecting adequate accelerometry data outside childcare (i.e., the home environment). For this reason, we decided after baseline assessments to only focus on collecting accelerometry data during childcare hours for the 6-month follow-up.

We found that there was a higher than expected turnover of staff and of children. This was largely due to the transient nature of the populations in some of the regional and rural communities. This resulted in the need to conduct additional training in many of the centres and taking longer to implement all components of the intervention. Centres were also unable to get any participation from families in the Jump Home component, with only a small number of parents (< 10) from a small number of centres (~ 3) reporting completion of the weekly challenges. Staff indicated that it was not unusual to have low levels of parental support for centre-based activities and this demonstrates the difficulties in working with families in these settings. The low levels of implementation of the home component resulted in physical activity not being assessed in the home environment at 6-month follow-up.

## Conclusions

The 6-month effects of the Jump Start interventions showed no difference in physical activity between children in the intervention and control centres. At this initial time point, this is due to low levels of implementation among intervention centres. This reinforces the importance of supporting centres to achieve high levels of fidelity and overcome barriers to implementation. It can take time in these settings for changes to be embedded into everyday routines and ongoing professional development is critical given the dynamic nature of these environments in these communities.

## Data Availability

Data supporting the results reported in this article are stored at the University of Wollongong. These data are available upon request by contacting the first author.
